# New Markers for Cardiovascular Disease in Psoriatic Patients: Preliminary Study on Monocyte Phenotype, ADAMTS7, and mTOR Activity

**DOI:** 10.3390/metabo13010116

**Published:** 2023-01-11

**Authors:** Khanty Loyola, Claudio Karsulovic, Raúl Cabrera, Claudio Perez, Lía Hojman

**Affiliations:** 1 Dermatology Section, Surgery Department, Facultad de Medicina Clínica Alemana de Santiago, Universidad del Desarrollo, Santiago P.O. Box 7630000, Chile; 2 Rheumatology Section, Internal Medicine Department, Facultad de Medicina Clínica Alemana de Santiago, Universidad del Desarrollo, Santiago P.O. Box 7630000, Chile; 3 Investigation in Dermatology and Autoimmunity—IDeA Lab, Instituto de Ciencias e Innovación en Medicina, Universidad del Desarrollo, Santiago P.O. Box 7630000, Chile; 4 Cancer Immunology and Regulation Laboratory, Immunology Disciplinary Program, Facultad de Medicina, Universidad de Chile, Santiago PO Box 8380000, Chile; 5 Cell Therapy Laboratory, Hospital Clínico de la Universidad de Chile, Santiago P.O. Box 8380000, Chile

**Keywords:** psoriasis, ADAMTS7, mTOR

## Abstract

Psoriasis is a skin disease with occasional involvement of non-cutaneous territories. Beyond the usual, cardiovascular events are more frequent in these patients and correlate only partially with disease activity, suggesting the presence of other unknown factors. We selected ten psoriatic patients without treatment in the last year and matched them for age and gender with eleven healthy subjects. Ficoll-extracted mononuclear cells were analyzed with flow cytometry for monocyte surface phenotype markers, intracellular NFκB/inflammasome-dependent interleukins, and chemotaxis receptor CXCR3. Using ELISA, patient serum was evaluated for ADAMTS7 and CXCL10. Inflammatory M1 monocytes showed higher levels of IL-1β and IL-6 in psoriatic patients. M2 monocytes also showed higher levels of intracellular inflammatory cytokines. Nevertheless, IL-6 values were higher compared to other monocytes and IL-1β. The mTORC activation markers ADAMTS7 and S6Rp were higher in psoriatic patients than in healthy controls. In psoriatic patients, serum levels of ADAMTS7 were elevated, and M2 monocytes showed a distinct inflammatory response with higher relative levels of NFκB-dependent IL-6 and less activity of the CXCR3–CXCL10 chemotactic pathway. These data suggest pathways with potential markers for prediction and early detection of cardiovascular risk in psoriatic patients.

## 1. Introduction

Psoriasis is a systemic disease with occasional comorbidities involving joints, enthesis, eyes, and gastrointestinal territories. Nonetheless, cardiovascular disease remains a major cause of death in these patients [1]. The severity of the disease has been considered as a predictor of extra-cutaneous involvement; however, particularly in accelerated atheromatosis and cardiovascular risk, increased frequency of Framingham traditional cardiovascular risk factors and higher clinical inflammation measured as Psoriasis Area Severity Index (PASI) are not able to accurately predict major cardiovascular events (MACE) in psoriatic patients [2]. Multiple attempts to better predict cardiovascular risk in these patients have been proposed [3]. Other factors are being studied to narrow the gap between predicted and actual incidence of cardiovascular events [4].

In psoriasis pathophysiology, Th1 and Th17 pathway activation could be associated with accelerated atheromatosis by multiple mechanisms [5]; moreover, which modules are involved in specific aspects of vessel wall changes have yet to be fully elucidated [5]. The mammalian adaptation complex (mTORC) also has an important role in the pathogenesis of the atherosclerosis process. mTORC inhibition is a therapeutic tool after angioplasty [6]. Moreover, mTORC interacts with inflammatory and matrix-changing proteins such as the transcription factor NFκB [7], and Disintegrin, and Metalloproteinase with Thrombospondin Motifs 7 (ADAMTS7) [8]. The ADAMTS family member, ADAMTSL5, has already been associated with psoriasis and its musculoskeletal manifestations, showing an association between psoriatic arthritis and ADAMTSL5 antibodies. Since ADAMTS7 has been described as an emerging factor in an accelerated atheromatous, measuring its levels in psoriatic patients is of great interest [9]. On the other hand, activation of the mTORC1 pathway, which targets the phosphorylation of multiple proteins, plays a critical part in monocyte/macrophage inflammatory phenotyping [10]. These cells actively participate in the atheromatous plaque formation and expansion [11]. ADAMTS7 is secreted by macrophages and monocytes and interacts with extracellular matrix proteins, changing blood vessel wall structure and creating conditions for unstable atheromatous plaques [12]. How mTORC activation and ADAMTS7 levels behave in psoriatic patients has, to date, not been published.

## 2. Materials and Methods

### 2.1. Study Population

Patient recruitment was conducted among those attending the Dermatology Clinic of Hospital Padre Hurtado. Only systemic treatment-naïve patients were selected. Ten patients underwent a full dermatological consultation by two board-certificate dermatologists, including a PASI assessment and complete clinical history. To maximize differences in this exploratory study, we selected patients with severe psoriasis, defined for our singular population as those with a PASI above 15. Blood samples were immediately processed using the mononuclear Ficoll (BD Biosciences, Franklin Lakes, NJ, USA) extraction procedure and frozen until analysis. In the second stage, we selected 11 age- and gender-matched healthy controls. Patient clinical features are shown in Table 1. All participants provided informed consent following the Declaration of Helsinki. The protocol was approved by the Institutional Review Board of Clínica Alemana de Santiago/Hospital Padre Hurtado (Acta 2020-79).

### 2.2. PBMC Extraction and Flow Cytometry

Venous blood (30 mL) was obtained by cubital venipuncture from all participants using BD Vacutainer 10 mL green cap heparin tubes (BD Biosciences, Franklin Lakes, NJ, USA). PBMC Ficoll extraction was performed. Cells were washed, followed by staining with Cell Viability Kit (BD Biosciences, Franklin Lakes, NJ, USA) and LUNA cell counter (Brightfield—Logos Biosystems, Anyang-si, South Korea) with cell viability of 96.5 ± 1.5%. PBMC were stained in duplicate with the following antibodies: Live-Dead-BUV496, V711-anti-CD3, FITC-A-anti-CD14, APC-Cy7-anti CD16, PERCP-Cy5.5-anti-CD163, APC-A-anti-CD163, BV605-Anti Human CCR2, BV786 anti-human HLA-DR, and A700-Anti-CXCR3 (BioLegend, San Diego, CA, USA) at room temperature for 30 min. Finally, we fixed and permeabilized cells using a BD Cytofix/Cytoperm fixation/permeabilization kit (BD Biosciences, Franklin Lakes, NJ, USA) and stained them with Pacific Blue-anti-IL-1β, PE-Cy7-Anti Human IL-6, (BioLegend, San Diego, CA, USA) and A647-Phospho-S6 Ribosomal Protein (Ser235/236) Antibody (Cell Signaling Technology, Danvers, MA, USA). Monocyte subset frequency and median fluorescence intensity were assessed on a FACS LSRFortessa instrument (BD Biosciences, Franklin Lakes, NJ, USA). The data were analyzed with FlowJo software (v10.7; TreeStar; Ashland, OR, USA).

### 2.3. ELISA Assay

ADAMTS7 and CXCL10 levels were measured using ELISA (Human ELISA-kit (colorimetric), Novus Biological—Bio-Techne, Denver, CO, USA) (ELISA kit, anti-CXCL10, and anti-IL-18, (BD Biosciences, Franklin Lakes, NJ, USA).

### 2.4. Flow Cytometry and Gating Strategy

**Figure 1:**
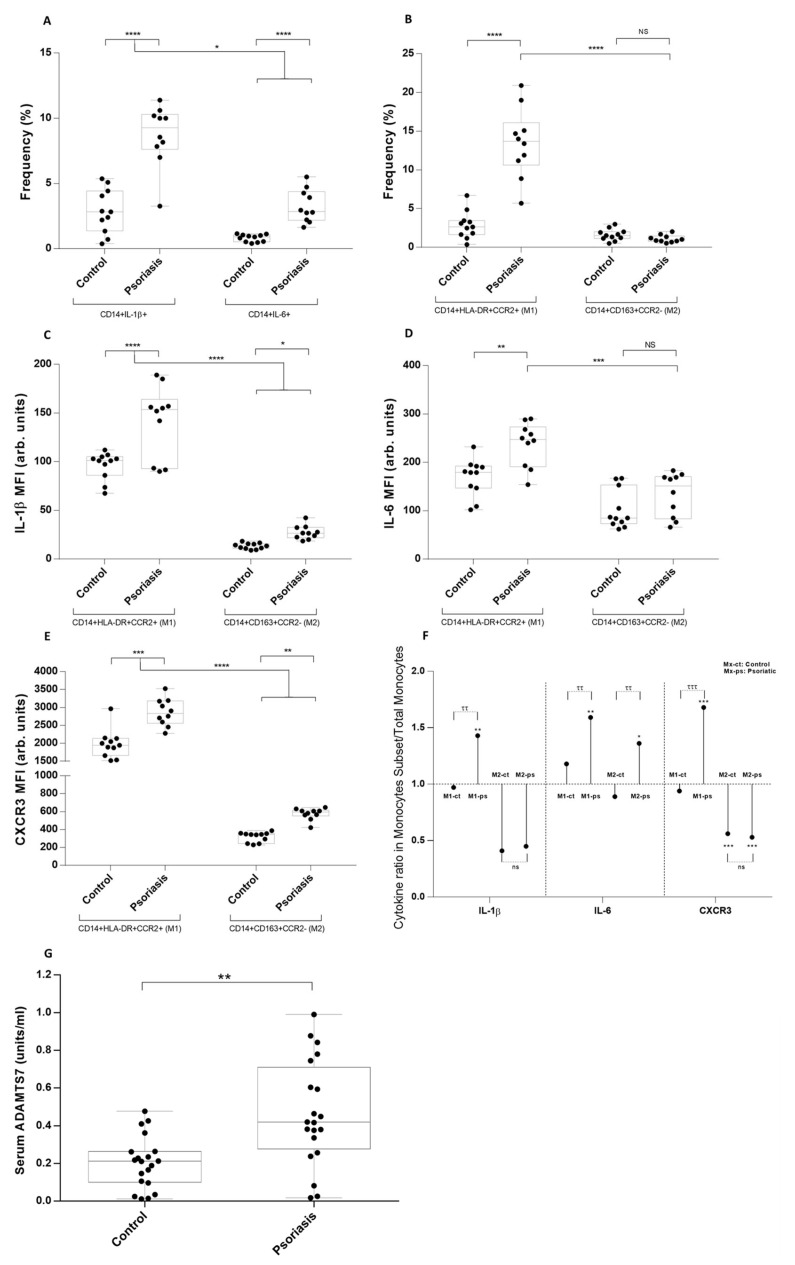
Monocyte phenotype, intracellular cytokine levels, and serum ADAMTS7 values in M1, M2, and total monocytes of psoriatic patients and healthy subjects. (**A**) Frequency of CD14+IL-1β+ and CD14+IL-6+ cells in psoriatic patients and healthy subjects. (**B**) Frequency of M1 and M2 monocytes in psoriatic patients and healthy subjects. (**C**) IL-1β mean fluorescence intensity (MFI) intracellular levels in M1 and M2 monocytes. (**D**) IL-6 MFI intracellular levels in M1 and M2 monocytes. (**E**) Chemotactic CXCR3 receptor MFI levels in M1 and M2 monocytes. (**F**) Ratio between MFI for IL-1β, IL-6, and CXCR3 in M1 and M2 monocytes versus total CD14+ monocytes in psoriatic and control subjects. (**G**) ADAMST7 serum values in psoriatic patients and healthy subjects. (**A**–**D**) **** *p* = 0.0001, *** *p* = 0.001, ** *p* = 0.003, * *p* = 0.039; (**E**) **** *p* = 0.0001, *** *p* = 0.0008, ** *p* = 0.001; (**F**) *** *p* = 0.0001, ** *p* = 0.002, * *p* = 0.01, ττ *p* = 0.002, ττ *p* = 0.004, ττ *p* = 0.002, τττ *p* = 0.0001; (**G**) ** *p* = 0.002. Mann–Whitney test and Spearman’s rank correlation test were used.

Live cells were selected from the total mononuclear cells. The live cells were then negatively selected for CD3 (T lymphocytes). The remaining cells were subsequently classified using CD14, HLA-DR, CD163, and CCR2 as markers according to [13]. Two subpopulations were defined: CD14+HLA-DR+CCR2+ cells (as M1, inflammatory cells), and CD14+CD163+CCR2-cells (as M2, non-inflammatory cells), as shown in Figure 1A. Downsampling and tSNE analysis were performed using the FlowJo plug-in available at http://www.flowjo.com. (accessed on 10 August 2022).

### 2.5. Statistical Analysis

Variables are shown as mean and standard deviation values. Continuous variables are expressed as mean and range or standard deviation, and discrete variables as percentage distributions. Fisher’s exact test and two other tests were used to compare qualitative data, and test-test and analysis of variance test were used for quantitative data. The correlation between variables was analyzed by Spearman’s rank correlation test. All the analysis was carried out using GraphPad Prism version 6.01 software. PCA and HeatMap analysis was performed using XLSTAT version 2022.2.1 (Addinsoft, New York, NY, USA). A *p*-value of <0.05 was considered statistically significant.

## 3. Results

After gathering psoriasis-related clinical features, cardiovascular risk factor was assessed. No significant differences in age, sex, and cardiovascular risk factors were found (Table 1). First, an immunophenotype analysis was designed for peripheral monocytes using the previously described M1–M2 classification. CD14, CD163, CCR2, and HLA-DR markers allowed us to define M1 monocytes (CD14+HLADR+CCR2+) and M2 monocytes (CD14+CD163+CCR2-) (Supplementary Figure S1A). In these subsets and in the total CD14+ monocytes, we subsequently performed a cytokine characterization by measuring inflammasome-derived intracellular IL-1β, NF-kB-dependent intracellular inflammatory IL-6, and the chemotaxis axis by measuring surface CXCR3 receptor and serum CXCL10 chemokine.

IL-1β+ and IL-6+ cells among CD14+ monocytes were three times higher in psoriatic patients than in the control group (Figure 1A). M1, but not M2, the frequency was higher in the psoriatic group (Figure 1B), and only M1 showed an elevated fluorescence for IL-1β (Figure 1C) and IL-6 (Figure 1D). Chemokine CXCR3 was elevated in psoriasis both in M1 and M2, with values six times higher in the first (Figure 1E). Further analysis showed that the difference between the IL-6 MFI ratio in M2/total CD14+ monocytes in psoriatic versus controls was higher than IL-1β in M2/total CD14+ monocytes, indicating that IL-6, despite being an inflammatory cytokine, does not behave in the same manner as IL-1β (Figure 1F). ADAMTS7 levels were analyzed in both groups; psoriatic patients had higher serum levels, which have not been previously reported (Figure 1G). 

**Figure 2:**
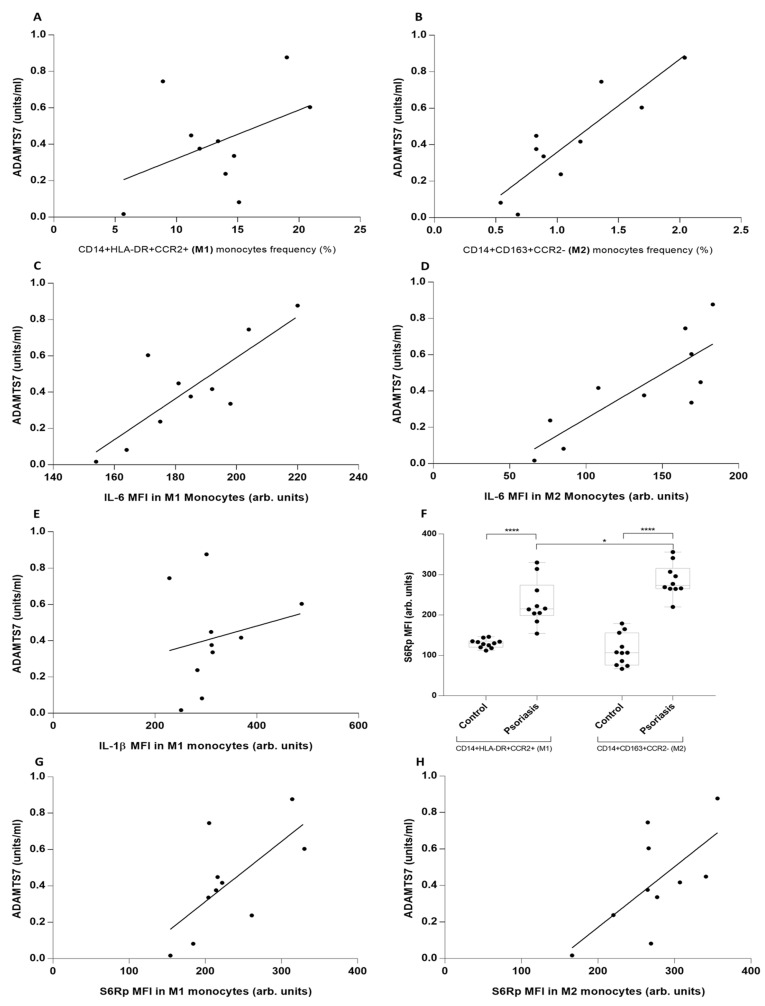
Correlation of serum ADAMTS7, IL-18, and CXCL10 levels with inflammatory cytokines, mTOR, and monocyte phenotype in psoriatic patients. (**A**,**B**) Correlation between serum ADAMTS7 level and M1 and M2 monocyte frequency measured by flow cytometry in psoriatic patients. (**C**,**D**) Correlation between serum ADAMTS7 IL-6 MFI levels in M1 and M2 monocytes measured by flow cytometry. (**E**) Correlation between serum ADAMTS7 levels of IL-1β MFI levels in M1 monocytes measured by flow cytometry. (**F**) S6Rp MFI in M1 and M2 monocytes from psoriatic patients. (**G**,**H**) Correlation of serum ADAMTS7 levels in psoriatic patients in M1 and M2 frequency of monocytes, by flow cytometry. (**A**) r = 0.43/*p* = 0.20, (**B**) r = 0.87/*p* = 0.0008, (**C**) r = 0.81/*p* = 0.0035, (**D**) r = 0.82/*p* = 0.0036, (**E**) r = 0.2/*p* = 0.56, (**F**) **** *p* = 0.0001, * *p* = 0.03, (**G**) r = 0.67/*p* = 0.033, (**H**) r = 0.63/*p* = 0.031. Mann–Whitney test and Spearman’s rank correlation test were used.

To better understand this unusual behavior of IL-6 in psoriasis, we performed a dimensional reduction using a flow-cytometry approach with tSNE and found that the two subpopulations of monocytes were perfectly differentiated, and its cytokine signature confirmed steady IL-6 values in the M2 subset from psoriatic patients (Supplementary Figure S1B). We further confirmed this observation by mathematical dimensional reduction of our variables using Principal Component Analysis. IL-6 MFI in M2 monocytes showed an orthogonal vectorial direction for both groups of patients in the PC1–PC2 biplot (Supplementary Figure S1C). Additional correlation analysis showed that only M2 monocytes correlate with ADAMTS7 serum levels (Figure 2A,B) and IL-6 (Figure 2C,D), but there is no correlation with IL-1β MFI levels in psoriasis (Figure 2E). Inflammasome-dependent cytokines IL-18 and IL-1β and CXCL10 correlate with M1 monocytes but not M2 monocytes (results not shown). At this point, IL-6 cytokine in M2 monocytes appears to correlate better with cardiovascular risk marker ADAMTS7. Since mTORC is a key participant in atheromatosis by modulating cell differentiation and phenotyping, we analyzed its activity via phosphorylation of S6R protein, a target of mTORC 1-protein synthesis pathways. S6Rp levels were higher in psoriatic patients, particularly M2 monocytes (Figure 2F). Following this, knowing that IL-6 is an NFκB-dependent cytokine associated with ADAMTS7 production, we assessed this association and found a positive correlation in psoriasis (Figure 2G,H). Finally, we could not find a correlation between clinical psoriasis features and molecular variables, indicating that clinical evaluation does not fully assess some of the underlying inflammatory processes (Supplementary Figure S2A–J).

## 4. Discussion

Here, we presented an exploratory study in a non-treated setting of severe psoriatic patients where unknown immunological factors potentially related to cardiovascular risk could be overexpressed. Considering this, three essential elements in atherosclerotic plaque development were first assessed: macrophage recruitment and their inflammatory and phagocytic differentiation, activation of adaptive pathways such as mTORC that help with myofibroblast trans-differentiation, and the hemodynamic and endothelial changes associated with the inflammatory process itself. We analyzed the first two elements, looking for aspects in a monocyte phenotype that would suggest specific recruitment to the plaque and then activation of the mTORC pathway. This pathway was activated in the psoriatic group even when CVD and obesity were similar between groups, suggesting an inflammatory–immune-mediated activation. We demonstrated the presence of M2 monocytes with a different pattern of inflammatory activation than M1 monocytes and less activity of the CXCL10-CXCR3 chemotaxis pathway. Chemotaxis pathways have recently been described as an alternative way to diminish the trafficking of inflammatory cells to ingrowing plaques [13]. This reinforces the idea of better understanding the movement of these cells between different compartments as an important aspect of accelerated atheromatosis.

Besides higher mTORC1 activation, our monocytes exhibited another interesting feature: the dissociation of IL-1β and IL-6 in M2 monocytes; IL-6 remains at elevated basal values in psoriatic patients and is mathematically demonstrated to behave differently from other cytokines in M2 monocytes (Supplementary Figure S1). Given that IL-6 depends on the activation of NFκB and not an inflammasome such as IL-1β, the idea of an NFκB-dependent activation of adaptation pathways such as mTORC or ADAMTS7 overproduction could be proposed. In previous studies, IL-6 induces endothelial dysfunction upregulating adhesion molecules in vitro [14]. On the other hand, in line with our findings, mTOR modulation drives inflammatory changes in endothelial cells [15]. Despite this, it is an exploratory study with a limited number of patients and without specific pathway analysis in other research models, so a causal relationship cannot be established.

## 5. Conclusions

We analyzed a distinct group of patients who did not have the confounding effect of methotrexate, NSAIDs, or biological therapies, which allowed us to remove an important variable in the analysis. Despite this, the monocyte’s phenotype showed complex behavior with different features between each subset. Non-inflammatory cells showed distinct interleukin marks, which can influence their participation in atheromatosis. Further studies with a larger number of patients are necessary to clarify whether ADAMTS7, NFκB-dependent interleukins, M2 monocyte phenotype, or mTORC activity could be added as early markers of cardiovascular risk in psoriatic patients.

## Figures and Tables

**Table 1:** Clinical features of control and psoriatic groups.

	Group 1	Group 2	
	Healthy Subjects (N = 11)	Psoriasis (N = 10)	*p* Value
Demographic			
Age, *years ± SD*	36.4 ± 5.7	39.1 ± 13	*NS*
Men, *n*	6	6	
Female, *n*	5	4	
Psoriasis Characteristics			
Diagnosis, *years ± SD*	n/a	6.1 ± 9.1	
PASI, *score ± SD*	n/a	27.3 ± 9.1	
Extracutaneous Involvement, *n*			
Ungueal	n/a	8	
Articular	n/a	0	
Treatment, *n*			
In use	n/a	0	
Suspended + 12 months	n/a	2	
Biologics in use, *n*	n/a	0	
CVD Characteristics			
Myocardial Infarction, *n*	0	0	
Stroke, *n*	0	0	
Cardiovascular Risk Factors			
HBP, *n*	1	3	
Lipid Disorder, *n*	1	2	
Obesity, *n*	1	2	
Diabetes, *n*	0	0	
Tobacco use, *(Pack/year)* ± SD	0.46 ± 1.15	3.53 ± 3.79	*NS*
Medication			
Statins, *n*	1	1	

PASI: Psoriasis Area Severity Index. n/a: not applicable. *NS*: Non-significant

## Data Availability

The data presented in this study are available in article and supplementary material.

## Supplementary Materials

The following supporting information can be downloaded at: https://www.mdpi.com/article/10.3390/metabo13010116/s1, Figure S1: Surface, intracellular, and serum variables analysis performing dimensional reduction using t-SNE and Principal Component Analysis; Figure S2: Correlation analysis between clinical features, intracellular cytokine levels, serum ADAMTS7, and monocyte phenotype.
