# Supplementary material for: New Markers for Cardiovascular Disease in Psoriatic Patients: Preliminary Study on Monocyte Phenotype, ADAMTS7, and mTOR Activity
Source: Metabolites. 2023 Jan 11;13(1):116. doi: 10.3390/metabo13010116 (PMC9864195; doi:10.3390/metabo13010116)
Supplement: Supplementary file 1 [file metabolites-13-00116-s001.zip › metabolites-2142793-supplementary.pdf]

SUP. FIGURE 1

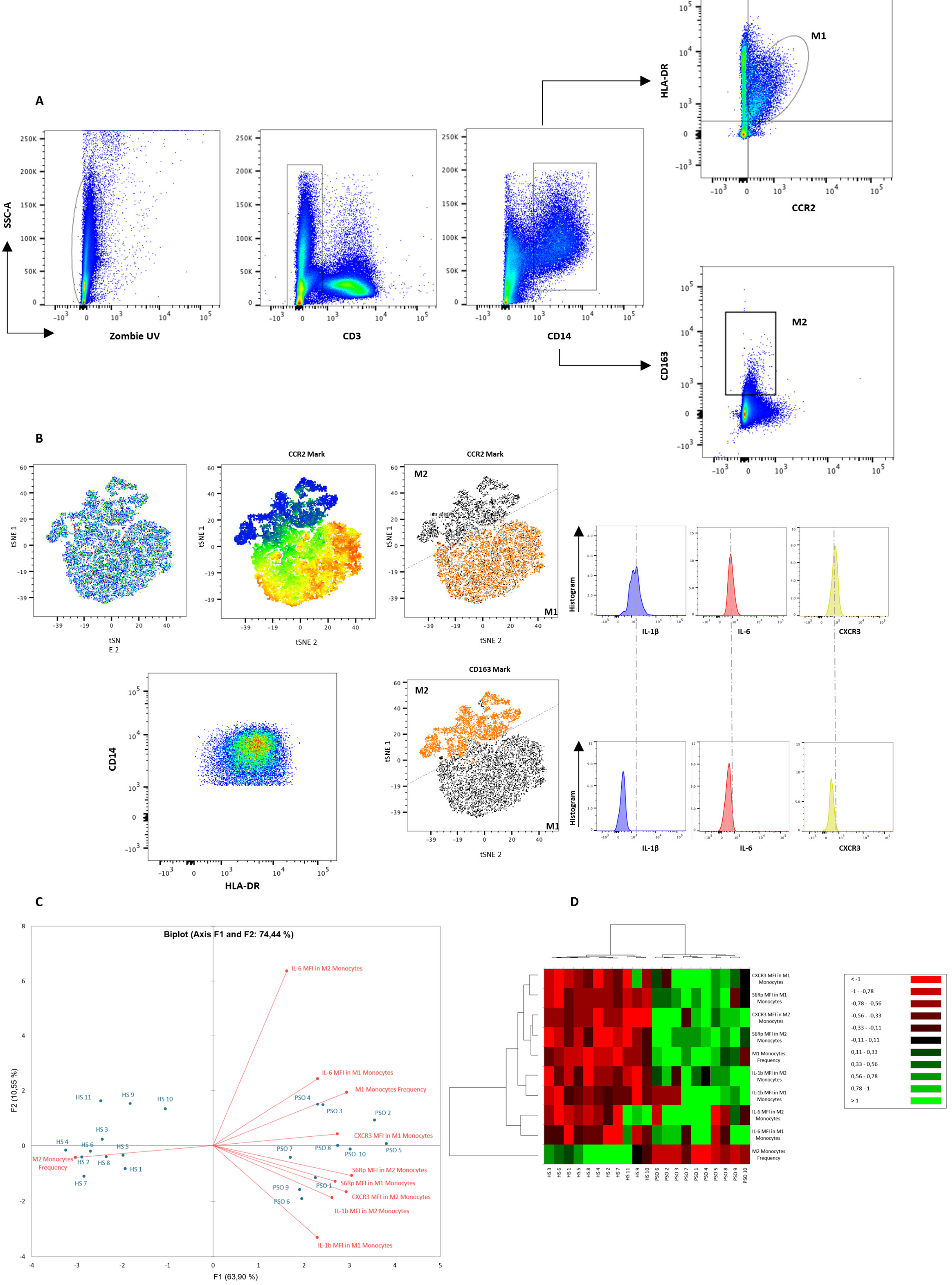
A
SSC-A
Zombie UV
CD3
CD14
HLA-DR
CCR2
M1
CD163
M2
B
tSNE 1
tSN
E 2
tSNE 2
CCR2 Mark
CD163 Mark
Histogram
IL-1β
IL-6
CXCR3
CD14
HLA-DR
C
Biplot (Axis F1 and F2: 74,44 %)
F1 (63,90 %)
F2 (10,55 %)
IL-6 MFI in M2 Monocytes
IL-6 MFI in M1 Monocytes
M1 Monocytes Frequency
CXCR3 MFI in M1 Monocytes
S6Rp MFI in M2 Monocytes
S6Rp MFI in M1 Monocytes
CXCR3 MFI in M2 Monocytes
IL-1b MFI in M2 Monocytes
IL-1b MFI in M1 Monocytes
M2 Monocytes
Frequency
HS 1
HS 2
HS 3
HS 4
HS 5
HS 6
HS 7
HS 8
HS 9
HS 10
HS 11
PSO 1
PSO 2
PSO 3
PSO 4
PSO 5
PSO 6
PSO 7
PSO 8
PSO 9
PSO 10
D
CXCR3 MFI in M1 Monocytes
S6Rp MFI in M1 Monocytes
CXCR3 MFI in M2 Monocytes
S6Rp MFI in M2 Monocytes
M1 Monocytes Frequency
IL-1b MFI in M2 Monocytes
IL-1b MFI in M1 Monocytes
IL-6 MFI in M2 Monocytes
IL-6 MFI in M1 Monocytes
M2 Monocytes Frequency
< -1
-1 - -0,78
-0,78 - -0,56
-0,56 - -0,33
-0,33 - -0,11
-0,11 - 0,11
0,11 - 0,33
0,33 - 0,56
0,56 - 0,78
0,78 - 1
> 1

Supplementary Figure S1: Surface, intracellular, and serum variables analysis performing dimensional reduction using t-SNE and Principal Component Analysis.

(A) Gating strategy from a representative psoriatic patient. Monocytes were first negatively selected for CD3 and subsequently selected among CD14+ cells for HLA-DR+CCR2+ in M1 and CD163+CCR2- M2 monocytes. (B) t-SNE simultaneously analyzes all variables, showing inflammatory markers in two identified distinctive subpopulations. (C) Biplot of all patients and variables and their co-localization in a PC1 vs. PC2 bidimensional graph. (D) Heatmap showing distribution and relative intensity of fluorescence values in all patients.

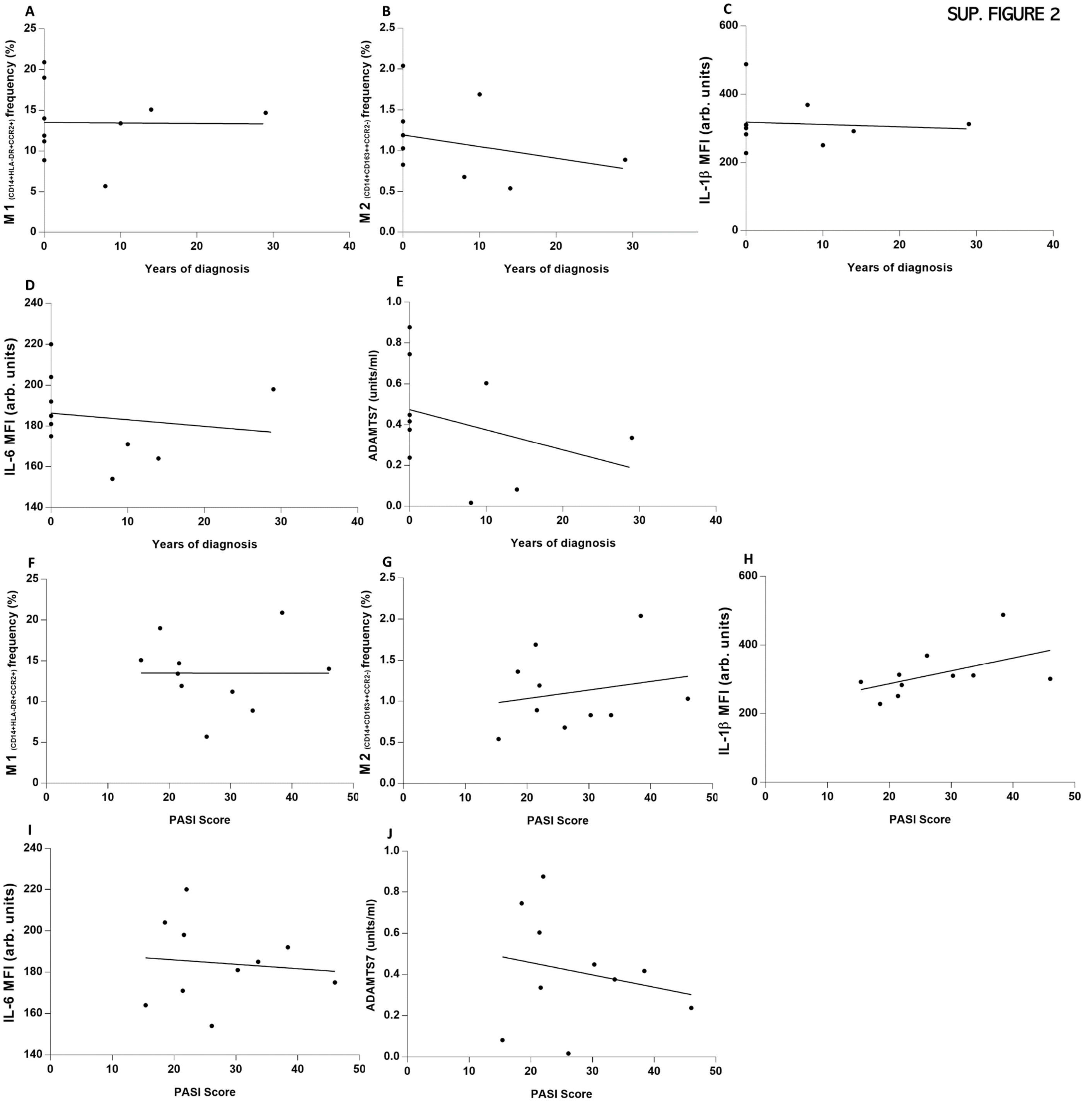

Supplementary Figure S2: Correlation analysis between clinical features, intracellular cytokine levels, serum ADAMTS7, and monocyte phenotype.

(A to E) Correlation between Years from Diagnosis and M1 and M2 frequency, IL-1β MFI, IL-6 MFI, and Serum ADAMTS7. (F to J) Correlation between PASI Score and M1 and M2 frequency, IL-1β MFI, IL-6 MFI, and Serum ADAMTS7. No correlations were found. Spearman's rank correlation tests were used.
